# University of Turku in the BioNLP'11 Shared Task

**DOI:** 10.1186/1471-2105-13-S11-S4

**Published:** 2012-06-26

**Authors:** Jari Björne, Filip Ginter, Tapio Salakoski

**Affiliations:** 1 Department of Information Technology, University of Turku, Turku Centre for Computer Science (TUCS), Joukahaisenkatu 3-5, 20520 Turku, Finland

## Abstract

**Background:**

We present a system for extracting biomedical events (detailed descriptions of biomolecular interactions) from research articles, developed for the BioNLP'11 Shared Task. Our goal is to develop a system easily adaptable to different event schemes, following the theme of the BioNLP'11 Shared Task: generalization, the extension of event extraction to varied biomedical domains. Our system extends our BioNLP'09 Shared Task winning Turku Event Extraction System, which uses support vector machines to first detect event-defining words, followed by detection of their relationships.

**Results:**

Our current system successfully predicts events for every domain case introduced in the BioNLP'11 Shared Task, being the only system to participate in all eight tasks and all of their subtasks, with best performance in four tasks. Following the Shared Task, we improve the system on the *Infectious Diseases *task from 42.57% to 53.87% F-score, bringing performance into line with the similar *GENIA Event Extraction *and *Epigenetics and Post-translational Modifications *tasks. We evaluate the machine learning performance of the system by calculating learning curves for all tasks, detecting areas where additional annotated data could be used to improve performance. Finally, we evaluate the use of system output on external articles as additional training data in a form of self-training.

**Conclusions:**

We show that the updated Turku Event Extraction System can easily be adapted to all presently available event extraction targets, with competitive performance in most tasks. The scope of the performance gains between the 2009 and 2011 BioNLP Shared Tasks indicates event extraction is still a new field requiring more work. We provide several analyses of event extraction methods and performance, highlighting potential future directions for continued development.

## Background

Biomedical event extraction is the process of automatically detecting statements of molecular interactions in research articles. Using natural language processing techniques, an event extraction system predicts relations between proteins/genes and the processes they take part in. Manually annotated corpora are used to evaluate event extraction techniques and to train machine-learning based systems.

Event extraction was popularised by the BioNLP'09 Shared Task on Event Extraction [1], providing a more detailed alternative for binary interaction extraction, where each pair of named entities (often protein names) co-occurring in the text is classified as interacting or not. Events extend this formalism by adding to the relations *direction, type *and *nesting*. Events define the type of interaction, such as *phosphorylation*, and commonly mark in the text a *trigger word *(e.g. "phosphorylates") describing the interaction. Directed events can define the role of their arguments as e.g. *cause *or *theme*, the agent or the target of the biological process. Finally, events can act as arguments of other events, creating complex nested structures that accurately describe the biological interactions stated in the text. For example, in the case of a sentence stating "Stat3 phosphorylation is regulated by Vav", a *phosphorylation*-event would itself be the argument of a *regulation*-event.

We developed for the BioNLP'09 Shared Task the Turku Event Extraction System, achieving the best performance at 51.95% F-score [2]. This system separated event extraction into multiple classification tasks, detecting individually the trigger words defining events, and the arguments that describe which proteins or genes take part in these events. Other approaches used in the Shared Task included e.g. joint inference [3]. An overall notable trend was the use of full dependency parsing [4-6].

In the following years, event extraction has been the subject of continuous development. In 2009, after the BioNLP'09 Shared Task, we extended our system and improved its performance to 52.85% [7]. In 2010, the system introduced by Miwa et. al. reached a new record performance of 56.00% [8].

In 2010, we applied the Turku Event Extraction System to detecting events in all 18 million PubMed abstracts, showing its scalability and generalizability into real-world data beyond domain corpora [9]. To facilitate the ease of use and applications based on the dataset, it has been transferred to the EVEX database, which also adds several layers of analysis to the data [10].

Participating in the BioNLP 2011 Shared Task [11,12], we have demonstrated the generalizability of the Turku Event Extraction System to different event extraction tasks by applying what is, to a large extent, the same system to every single task and subtask. Following the Shared Task, we now further improve performance on the ID (Infectious Diseases) task, provide a detailed analysis of performance on different corpora with learning curves, and evaluate the suitability of events from the EVEX database for use as additional training data.

## Methods

**Figure 1:**
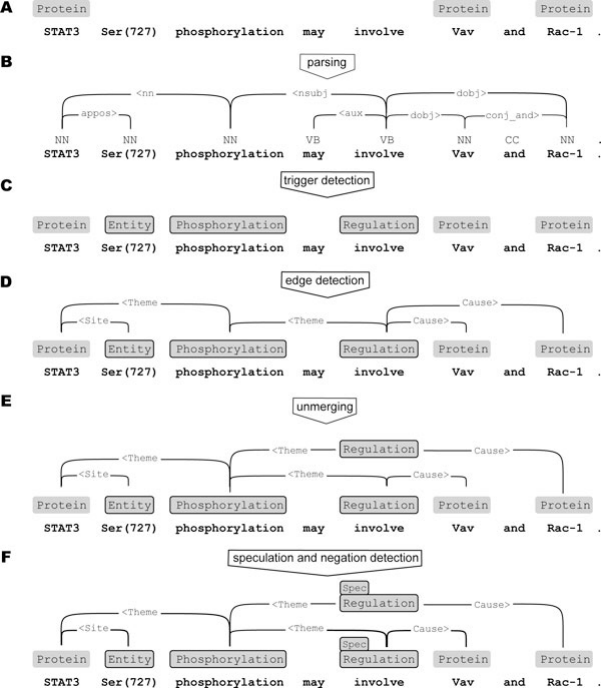
**Event extraction**. In most tasks named entities are given (A). Sentences are parsed (B) to produce a dependency parse. Entities not given are predicted through trigger detection (C). Edge detection predicts event arguments between entities (D) and unmerging creates events (E). Finally, event modality is predicted (F). When the graph is converted to the Shared Task format, site arguments are paired with core arguments that have the same target protein.

Our system divides event extraction into three main steps (Figure 1C, D and 1E). First, entities are predicted for each word in a sentence. Then, arguments are predicted between entities. Finally, entity/argument sets are separated into individual events.

### Graph representation

The BioNLP'11 Shared Task consists of eight separate tasks. Most of these follow the BioNLP'09 Shared Task annotation scheme, which defines events as having a trigger entity and one or more arguments that link to other events or protein/gene entities. This annotation can be represented as a graph, with trigger and protein/gene entities as nodes, and arguments (e.g. *theme*) as edges. In our graph representation, an event is defined implicitly as a trigger node and its outgoing edges (see Figure 1F).

Most of the BioNLP'11 Shared Task tasks define task-specific annotation terminology, but largely follow the BioNLP'09 definition of events. Some new annotation schemes, such as the bracket notation for protein references in the CO (Protein/Gene Coreference) task can be viewed simply as alternative representations of arguments. The major new feature is *relations *or *triggerless events*, used in the REL (Entity Relations), REN (Bacteria Gene Renaming), BB (Bacteria Biotopes) and BI (Bacteria Gene Interactions) tasks. In our graph representation, this type of event is a single, directed edge.

**Figure 2:**
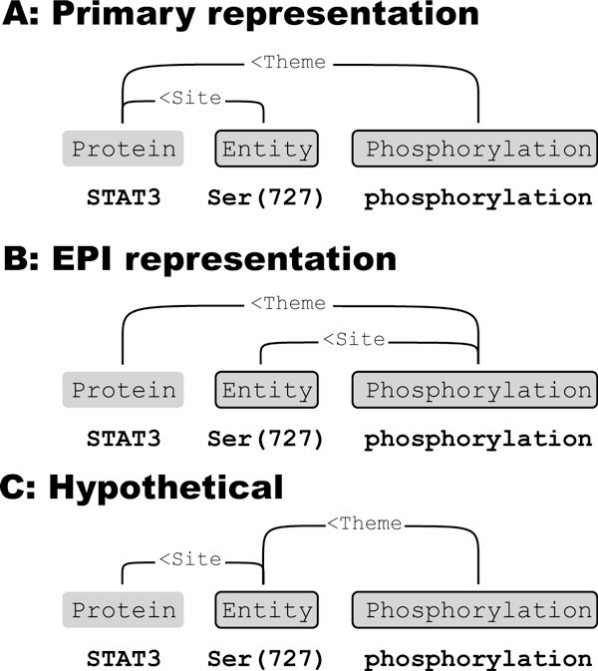
**Site argument representation**. Site arguments add detail to core arguments, and each site argument is paired with one core argument. (A) In most tasks we link both core and site arguments to given protein nodes. This minimizes the number of outgoing edges per trigger node, simplifying unmerging, but loses the connection between site and core arguments. (B) In the EPI task, all events with site-arguments have a single core argument, so linking sites to the trigger node preserves the site/core connection. (C) To both limit number of arguments in trigger nodes and preserve site information, event arguments using sites could be linked to protein nodes through the site entity. However, in this approach the core argument would remain undetected if the site wasn't detected.

Some event arguments have a matching *site *argument that determines the part of the protein the argument refers to (Figure 2). To allow detection of core arguments independently of site arguments, in most tasks we link both core and site arguments directly to proteins (Figure 2A and 2C). Connecting site arguments to the protein instead of the event also reduces the number of outgoing edges per predicted event, simplifying unmerging (see section *Unmerging*). However, if several events' core arguments refer to the same protein, the matching of site arguments to core arguments becomes ambiguous, limiting performance on site argument detection, but in most cases maximizing the performance on the core task is preferable.

**Figure 3:**
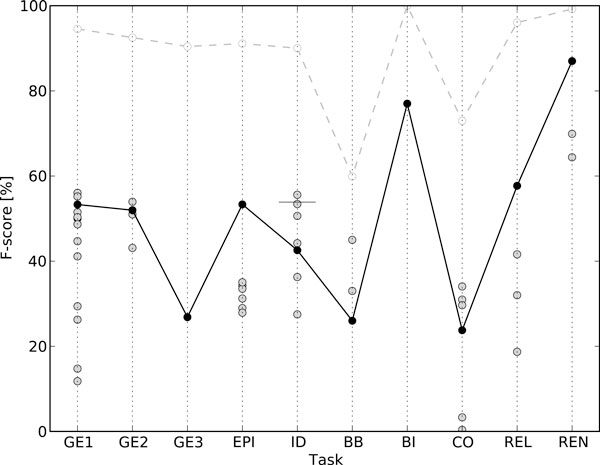
**Ranking of the systems participating in the BioNLP'11 Shared Task**. Our system is marked with black dots and the dotted line shows its theoretical maximum performance (see section *Graph representation*) with all correct classifications. The horizontal line in ID results shows the improved, post Shared Task result.

To further simplify event extraction all sentences are processed in isolation, so events crossing sentence boundaries (intersentence events, Table 1) cannot be detected. This also limits the theoretical maximum performance of the system (see Figure 3).

**Table 1:** Corpus statistics

Corpus	Sentences	Events	Equiv events	Nesting events	Intersentence events	Neg/spec events
GE'09	8906	11285	7.9%	38.8%	6.0%	12.1%
GE	11581	14496	6.6%	37.2%	6.0%	13.3%
EPI	7648	2684	9.1%	10.2%	9.3%	10.1%
ID	3193	2931	5.3%	21.3%	3.9%	4.9%
BB	1762	5843	79.4%	N/A	86.0%	0%
BI	120	458	0%	N/A	0%	0%
CO	8906	5284	0%	N/A	8.5%	N/A
REL	8906	2440	4.2%	N/A	0%	0%
REN	13235	373	0%	N/A	2.4%	0%

Numbers are for all available annotated data, i.e. the merged training and development sets. Event numbers include the resolved equivalencies.

In the provided data an event is annotated only once for a set of equivalent proteins. For example, in the sentence "Ubiquitination of caspase 8 (casp8)" a *ubiquitination *event would be annotated only for "caspase 8", "casp8" being marked as equivalent to "caspase 8". To improve training data consistency, our system fully resolves these equivalences into new events, also recursively when a duplicated event is nested in another event (Table 1). Resolved equivalences were used for event extraction in the BioNLP'11 GE (GENIA Event Extraction), ID (Infectious Diseases), EPI (Epigenetics and Post-translational Modifications) and BB (Bacteria Biotopes) tasks, although based on tests with the GE dataset their impact on performance was negligible.

### Machine learning

The machine learning based event detection components classify examples into one of the positive classes or as negatives, based on a feature vector representation of the data. To make these classifications, we use the SVM^*multiclass *^support vector machine [13,14] with a linear kernel. An SVM must be optimized for each classification task by experimentally determining the regularization parameter C. This is done by training the system on a training dataset, and testing a number of C values on a development dataset. When producing predictions for the test set, the classifier is retrained with combined training and development sets, and the test data is classified with the previously determined optimal value of C.

In the BioNLP'09 Shared Task we optimized the three main parameters (trigger-detector, recall-adjustment and edge-detector) in an exhaustive grid search against the final metric. Due to time constraints, for the BioNLP'11 Shared Task, only the recall-adjustment parameter (see section *Trigger Detection*) was optimized against the final metric, edge and trigger detector parameters being optimized in isolation.

Following the Shared Task, we tested again the three-parameter grid search for the GE, EPI and ID tasks. Performance differences were negligible, so with the current system and feature representations we can assume that optimizing trigger and edge detector regularization parameters in isolation produces SVM models applicable for the overall task.

### Syntactic analyses

The machine learning features that are used in event detection are mostly derived from the syntactic parses of the sentences. Parsing links together related words that may be distant in their linear order, creating a parse tree (see Figure 1B).

We used the Charniak-Johnson parser [15] with David McClosky's biomodel [16] trained on the GENIA corpus and unlabeled PubMed articles. The parse trees produced by the Charniak-Johnson parser were further processed with the Stanford conversion tool [17], creating a dependency parse [18].

In the supporting tasks (REL, REN and CO) this parsing was done by us, but in the main tasks the organizers provided official parses which were used [19]. All parses for tasks where named entities were given as gold data were further processed with a *protein name splitter *that divides at punctuation tokens which contain named entities, such as "p50/p65" or "GATA3-binding", which would otherwise lead to multiple entities or triggers having the same head token, preventing detection of events between them.

### Feature groups

To convert text into features understood by the classifier, a number of analyses are performed on the sentences, mostly resulting in binary features stating the presence or absence of some attribute. Basic features such as token texts can also be combined into more specific features, such as the *N*-grams used in edge detection.

**Token features **can be generated for each word token, and they define the text of the token, its Porter-stem [20], its Penn Treebank part-of-speech-tag, character bi-and trigrams, presence of punctuation or numeric characters etc.

**Sentence features **define the number of named entities in the sentence as well as bag-of-words counts for all words.

**Dependency chains **follow the syntactic dependencies up to a depth of three, starting from a token of interest. They are used to define the immediate context of these words.

**Dependency path *N*-grams **are built from the shortest undirected path of tokens and dependencies linking together two entities, and are used in edge detection. *N*-grams join together a token with its two flanking dependencies as well as each dependency with its two flanking tokens. Each token or dependency has a number of attributes such as text or type, which are joined with the attributes of its neighbours to form the *N*-gram. While these *N*-grams follow the direction of the entire path, the governor-dependent directions of individual dependencies are used to define additional token bigrams.

**Trigger features **can be built for trigger or entity nodes already present, i.e. the given gold entities, and also predicted triggers when doing edge detection and unmerging. These features include the types and supertypes (the *GeneEntity *and *ProteinEntity *in the BI task) of the trigger or entity nodes, and combinations thereof.

**External features **are additional features based on data external to the corpus being processed. Such features can include e.g. the presence of a word in a list of key terms, Wordnet hypernyms, or other resources that enhance performance on a particular task. These are described in detail in section *Results and discussion*.

### Trigger Detection

Trigger words are detected by classifying each token as negative or as one of the positive trigger classes. Sometimes several triggers overlap, in which case a merged class (e.g. *phosphorylation-regulation*) is used. Such cases are quite rare, for example in the GENIA corpus development set only 1.6% (44 out of 2741) of positive trigger examples belong to a merged class. After trigger prediction, triggers of merged classes are split into their component classes. In practice, examples of merged classes are rarely predicted, except for the most common overlapping classes.

Most tasks evaluate trigger detection using approximate span, so detecting a single token is enough. However, this token must be chosen consistently for the classifier to be able to make accurate predictions. For multi-token triggers, we select as the trigger word the *syntactic head*, the root token of the dependency parse subtree covering the entity.

When optimizing the SVM C-parameter for trigger and edge detection, it is optimized in isolation, maximizing the F-score for that classification task. Edges can be predicted for an event only if its trigger has been detected, but often the C-parameter that maximizes trigger detection F-score has too low recall for optimal edge detection. A *recall adjustment *step is used to fit together the trigger and edge detectors. For each example, the classifier gives a confidence score for each potential class, and picks as the predicted class the one with the highest score. In recall adjustment, the confidence score of each negative example is multiplied with a multiplier, and if the result falls below the score of another class, that class becomes the new classification. This multiplier is determined experimentally by optimizing against overall system performance, using the official task metric if a downloadable evaluator is available (GE, BB, REL, REN and CO in the Shared Task, EPI and ID evaluators have been published since then), or edge detection F-score if there isn't one.

### Edge detection

Edge detection is used to predict event arguments or triggerless events and relations, all of which are defined as edges in the graph representation. The edge detector defines one example per direction for each pair of entities in the sentence, and uses the SVM classifier to classify the examples as negatives or as belonging to one of the positive classes. As with the trigger detector, overlapping positive classes are predicted through merged classes (e.g. *cause-theme*). There are usually fewer edge types than trigger types, so merged classes are even less common than in trigger detection, for example in the GENIA corpus development set only 5 out of 3634 positive edge examples belong to a merged class. Task-specific rules defining valid argument types for each entity type are used to considerably reduce the number of examples that can only be negatives.

### Unmerging

In the graph representation, events are defined through their trigger word node, resulting in overlapping nodes for overlapping events. The trigger detector can however predict a maximum of one trigger node per type for each token. When edges are predicted between these nodes, the result is a *merged graph *where overlapping events are merged into a single node and its set of outgoing edges. Taking into account the limits of trigger prediction, the edge detector is also trained on a merged graph version of the gold data.

To produce the final events, these merged nodes need to be "pulled apart" into valid trigger and argument combinations. In the BioNLP'09 Shared Task, this was done with a rule-based system. Since then, further research has been done on machine learning approaches for this question [21,22]. In our current system, unmerging is done as an SVM-classification step. An example is constructed for each argument edge combination of each predicted node, and classified as a true event or a false event to be removed. Tested on the BioNLP'09 Shared Task data, this system performs roughly on par with our earlier rule-based system, but has the advantage of being more general and thus applicable to all BioNLP'11 Shared Task tasks. The unmerging step is not required for *triggerless events *which are defined by a single edge.

All of the tasks define varied, detailed limits on valid event type and argument combinations. A final validation step based on task-specific rules is used to remove structurally incorrect events left over from preceding machine learning steps. For example, for the GENIA corpus development set, this validation step removed 6.2% of the predicted events which did not conform to task specific structural requirements.

### Modality detection

Speculation and negation are detected independently, with binary classification of trigger nodes. The features used are mostly the same as for trigger detection, with the addition of a list of speculation-related words selected manually from the BioNLP'09 ST corpus.

## Results and discussion

The BioNLP'11 Shared Task consists of five main tasks and three supporting tasks (Table 2). Additionally, many of these tasks specify separate subtasks. Except for the GE-task, which defines three main evaluation criteria, all tasks have a single primary evaluation criterion. All evaluations are based on F-score, the harmonic mean of precision and recall. Performance of all systems participating in the BioNLP'11 Shared Task is shown in Figure 3. Our system's performance on both development and test sets of all tasks is shown in Table 3.

**Table 2:** Event types

Event type	Corpora	Core arguments	Optional arguments
Gene expression	GE, ID	Theme(Protein, Regulon/Operon^ID^)	
Transcription	GE, ID	Theme(Protein, Regulon/Operon^ID^)	
Protein catabolism	GE, ID	Theme(Protein)	
Phosphorylation*	GE, EPI, ID	Theme(Protein)	Site(Entity)
Localization	GE, ID, BB	Theme^GE, ID^(Protein, Core entity^ID^), Bacterium^BB^(Bacterium), Localization^BB^(Host, HostPart, Geographical, Environmental, Food, Medical, Soil, Water)	AtLoc^GE, ID^(Entity), ToLoc^GE, ID^(Entity)
Binding	GE, ID	Theme(Protein, Core entity^ID^)+	Site(Entity)+
Regulation	GE, ID	Theme(Protein, Core entity^ID^, Event), Cause(Core entity^ID^, Event)	Site(Entity), CSite(Entity)
Positive regulation	GE, ID	Theme(Protein, Core entity^ID^, Event), Cause(Protein, Core entity^ID^, Event)	Site(Entity), CSite(Entity)
Negative regulation	GE, ID	Theme(Protein, Core entity^ID^, Event), Cause(Core entity^ID^, Event)	Site(Entity), CSite(Entity)
Process	ID	Participant(Core entity)	
Hydroxylation*	EPI	Theme(Protein)	Site(Entity)
Ubiquitination*	EPI	Theme(Protein)	Site(Entity)
DNA methylation*	EPI	Theme(Protein)	Site(Entity)
Glycosylation*	EPI	Theme(Protein)	Site(Entity)
Acetylation*	EPI	Theme(Protein)	Site(Entity)
Methylation*	EPI	Theme(Protein)	Site(Entity)
Catalysis	EPI	Theme(Event), Cause(Protein)	
PartOf	BB	HostPart(HostPart), Host(Host)	
RegulonDependence	BI	Regulon(Regulon), Target(GeneEntity, ProteinEntity)	
BindTo	BI	Agent(ProteinEntity), Target(Site, Promoter, Gene, GeneComplex)	
TranscriptionFrom	BI	Transcription(Transcription, Expression), Site(Site, Promoter)	
RegulonMember	BI	Regulon(Regulon), Member(GeneEntity, ProteinEntity)	
SiteOf	BI	Site(Site), Entity(Site, Promoter, GeneEntity)	
TranscriptionBy	BI	Transcription(Transcription), Agent(ProteinEntity)	
PromoterOf	BI	Promoter(Promoter), Gene(GeneEntity, ProteinEntity)	
PromoterDependence	BI	Promoter(Promoter), Protein(GeneEntity, ProteinEntity)	
ActionTarget	BI	Action(Action, Expression, Transcription), Target(*Any type*)	
Interaction	BI	Agent(GeneEntity, ProteinEntity), Target(GeneEntity, ProteinEntity)	
Coref	CO	Anaphora(Exp), Antecedent(Exp), Reference(Protein)+	
Protein-Component	REL	Arg1(Protein), Arg2(Entity)	
Subunit-Complex	REL	Arg1(Protein), Arg2(Entity)	
Renaming	REN	Former(Gene), New(Gene)	

The event types for all tasks, their core arguments used for the primary evaluation and optional arguments for secondary evaluation. Superscripts show the arguments and targets limited to a specific task for events present in multiple tasks. Starred events have in the EPI task a corresponding reverse event (e.g. Dephosphorylation) with identical argument types. The plus-sign indicates where multiple arguments of the same type are allowed for one event.

**Table 3:** Devel and test results for the BioNLP'11 Shared Task

Corpus	Devel F	Test F
GE'09 task 1	56.27	53.15
GE'09 task 2	54.25	50.68

GE task 1	55.78	53.30
GE task 2	53.39	51.97
GE task 3	38.34	26.86
EPI	56.41	53.33
ID	44.92	42.57
BB	27.01	26
BI	77.24	77
CO	36.22	23.77
REL	65.99	57.7
REN	84.62	87.0

The performance of our new system on the BioNLP'09 ST GENIA dataset is shown for reference, with task 3 omitted due to a changed metric. For GE-tasks, the Approximate Span & Recursive matching criterion is used. In many tasks, the development and test set results differ considerably, which may be partially explained by noise unseen due to lack of cross-validation and by the event distribution not being stratified across the sets.

In this section we also describe the approaches required for adapting the system to the different tasks. The primary adaptation was addition of task specific feature sets, although the majority of features were shared between all tasks. In some tasks, such as EPI, the graph representation was slightly altered. As the Turku Event Extraction System deals only with nodes and edges, the modified graph representation affected the system primarily in conversion to or from the Shared Task format. Finally, in tasks where all entities and triggers were given, the event extraction process was started from the edge detection step. All in all, task specific requirements resulted in relatively little additional code, consisting mostly of specialized versions of the generic trigger and edge detection modules.

### GENIA (GE)

The GENIA task is the direct continuation of the BioNLP'09 Shared Task. The BioNLP'09 ST corpus consisted only of abstracts. The new version extends this data by 30% with full text PubMed Central articles [23].

Our system applied to the GE task is the most similar to the one we developed for the BioNLP'09 Shared Task. The major difference is the replacement of the rule-based unmerging component with an SVM based one.

The GE task has three subtasks, task 1 is detection of events with their main arguments, task 2 extends this to detection of sites defining the exact molecular location of interactions, and task 3 adds the detection of whether events are stated in a negated or speculative context.

For task 3, speculation and negation detection, we considered the GE, EPI and ID task corpora similar enough to train a single model on. Compared to training on GE alone, example classification F-score decreased for negation by 8 pp and increased for speculation by 4 pp. Overall task 3 processing was considerably simplified.

Our system placed third in task 1, second in task 2 and first in task 3. Task 1 had the most participants, making it the most useful for evaluating overall performance. Our F-score of 53.30% was within three percentage points of the best performing system (by team FAUST [24]), indicating that our chosen event detection approach still remains competitive. For reference, we ran our system also on the BioNLP'09 data, reaching an F-score of 53.15%, a slight increase over the 52.85% we have previously reached [7].

### Epigenetics and Post-translational Modifications (EPI)

All events in the EPI task that have additional arguments (comparable to the site-arguments in the GE-task) have a single core argument [25]. We therefore use for this task a slightly modified graph representation, where all additional arguments are treated as core arguments, linking directly to the event node (Figure 2B), thus preserving the core/site argument pairings. The number of argument combinations per predicted event node remains manageable for the unmerging system and full recovery of additional arguments is possible.

Eight of the EPI event types have corresponding reverse events, such as *phosphorylation *and *dephosphorylation*. Many of these reverse events are quite rare, resulting in too little training data for the trigger detector to find them. Therefore we merge each reverse event type into its corresponding forward event type. After trigger detection, an additional rule-based step separates them again. Most of the reverse classes are characterized by a "de"-prefix in their trigger word, so the types of all such triggers are negated, as are the types of triggers whose text contains one of the strings "remov", "loss" or "erasure". On the EPI training dataset, this rule-based step determined correctly whether an event was reversed in 99.6% of cases (1698 out of 1704 events). Using this approach, primary criterion F-score on the development set increased 1.33 percentage points from 55.08% to 56.41%. Several previously undetectable small reverse classes became detectable, with e.g. *deubiquitination *(8 instances in the development set) detected at 77.78% F-score.

Our system ranked first on the EPI task, outperforming the next-best system (team FAUST) by over 18 percentage points. On the alternative core metric our system was also the first, but the FAUST system was very close with only a 0.27 percentage point difference. Following the Shared Task, it was confirmed that we were the only team to attempt detection of non-core arguments, explaining the large difference to other systems on the full task [25].

### Infectious Diseases (ID)

The annotation scheme for the ID task closely follows the GE task, except for an additional *process *event type that may have no arguments, and for five different entity types in place of the *protein *type [26]. Our approach for the ID task was identical to the GE task, but performance relative to the other teams was considerably lower. Primary evaluation metric F-score was 42.57% vs. 43.44% for the core metric which disregards additional arguments, indicating that these were not the reason for low performance.

Following the Shared Task, we analysed the results to determine the causes of our system lagging behind on the ID task. Compared to other participants, performance was especially low on the *process *events. A closer analysis of the system revealed that our original implementation of the unmerging component did not consider triggers with zero arguments as candidates for events. Allowing these *process *triggers to form events improved performance to 50.72%.

In the Shared Task, the teams with better performance succesfully utilized the similarity of the ID and GE datasets. The three machine learning systems [24,27,28] were trained for the ID task on a combination of ID and GE data, while the rule-based Concordia system [29] was developed to have mostly a single rule set for the GE, EPI and ID tasks. Following these approaches, we added the GE corpus into the training data of the ID task trigger and edge detectors, further increasing performance to 53.87%.

Together, these improvements increased our primary criterion performance on the test set by 11.30 percentage points. Compared to the Shared Task results, our new results place us second, just 1.72 pp after the leading system.

The new performance of 53.87% is very close to our system's performance of 53.30% and 53.33% on the similar GE and EPI tasks, indicating that the system's generally high performing approach is now fully applied also to the ID task.

### Bacteria Biotopes (BB)

The BB task considers detection of events about bacteria and their habitats [30]. The task defines only two event types but a large number of entity types which fall into five supertypes. All entities must be predicted and all events are triggerless.

Unlike in the other main tasks, in the BB task exact spans are required for *Bacterium*-type entities, which usually consist of more than one token (e.g. *B. subtilis*). After trigger detection, a rule-based step attempts to extend predicted trigger spans to reach the correct span. Starting from the detected trigger head token, it extends the span forwards and backwards as long as each encountered token is a known bacterium name substring. These substrings are derived from the List of Prokaryotic names with Standing in Nomenclature [31,32]. About 20 additional rules select for tokens based on common bacteria suffixes (e.g. "um", "ans", "bacter", "plasma") and a further 16 rules select for other known bacterium substrings (e.g. "strain", "subspecies"). When extending the spans of BB training set gold entity head tokens, this step produced the correct span for 91% (399 out of 440) of *Bacterium*-type entities.

To aid in detecting *Bacterium*-entities the list of bacteria names from the List of Prokaryotic names with Standing in Nomenclature was used as external features, marking for each token as a binary feature whether it has been seen in a known bacterium name. To help in detecting the heterogeneous habitat-entities, synonyms and hypernyms from Wordnet were used [33]. The development set lacked some event classes, so we moved some documents from the training set to the development set to include these.

The best system in the BB task was by team Bibliome, with an F-score of 45% [34]. Our F-score of 26% was the lowest of the three participating systems, and detailed results show a consistently lower performance in detecting the entities. The large number of intersentence events (Table 1) also considerably limited performance (Figure 3).

### Bacteria Gene Interactions (BI)

The BI-task considers events related to genetic processes of the bacterium *Bacillus subtilis *[35]. This task defines a large number of both entity and event types, but all entities are given as gold-standard data, therefore we start from edge detection (Figure 1D). All BI events are triggerless.

In this task manually curated syntactic parses are provided. As also automated parses were available, we tested them as an alternative. With the Charniak-Johnson/McClosky parses overall performance was only 0.65 percentage points lower (76.59% vs. 77.24%). As with the BB task, we moved some documents from the training set to the development set to include missing classes.

Despite this task being very straightforward compared to the other tasks we were the only participant. Therefore, too many conclusions shouldn't be drawn from the performance, except to note that a rather high F-score is to be expected with all the entities being given as gold data.

### Protein/Gene Coreference (CO)

In the CO supporting task the goal is to extract anaphoric expressions [36]. Even though our event extraction system was not developed with coreference resolution in mind, the graph representation can be used for the coreference annotation, making coreference detection possible. *Anaphoras *and *Antecedents *are both represented as *Exp*-type entities, with *Coref*-type edges linking *Anaphora*-entities to *Antecedent*-entities and *Target*-type edges linking *Protein*-type entities to *Antecedent*-entities.

In the CO-task, character spans for detected entities must be in the range of a full span and minimum span. Therefore in this task we used an alternative trigger detector. Instead of predicting one trigger per token, this component predicted one trigger per each syntactic phrase created by the Charniak-Johnson parser. Since these phrases don't cover most of the CO-task triggers, they were further subdivided into additional phrases, e.g. by cutting away determiners and creating an extra phrase for each noun-token, with the aim of maximizing the number of included triggers and minimizing the number of candidates.

The best system in the CO task was by University of Utah, with a performance of 34.05% [37]. Our system placed fourth out of six, reaching an F-score of 23.77%. Coreference resolution being a new subject for us and our system not being developed for this domain, we consider this an encouraging result, but conclude that in general dedicated systems should be used for coreference resolution.

### Entity Relations (REL)

The REL supporting task concerns the detection of static relations, *Subunit-Complex *relations between individual proteins and protein complexes and *Protein-Component *relations between a gene or protein and its component, such as a protein domain or gene promoter [38]. In our graph representation these relations are defined as edges that link together given protein/gene names and *Entity*-type entities detected by the trigger detector.

To improve entity detection, additional features are used. Derived from the REL annotation, these features highlight structures typical for biomolecular components, such as aminoacids and their shorthand forms, domains, motifs, loci, termini and promoters. Many of the REL entities span multiple tokens. Since the trigger detector predicts one entity per token, additional features are defined to mark whether a token is part of a known multi-token name. The texts of the preceding tokens are joined together, and the presence of known multi-token triggers in this string are marked as features. The system still predicts only one token for each trigger, but can this way determine whether that token belongs to a known, larger trigger expression.

Our system had the best performance out of four participating systems with an F-score of 57.7%, over 16 percentage points higher than the next. Performance for the two event classes was quite close, 58.43% for Protein-Component and 56.23% for Subunit-Complex.

### Bacteria Gene Renaming (REN)

The REN supporting task is aimed at detecting statements of *B. Subtilis *gene renaming where a synonym is introduced for a gene [35]. The REN task defines a single relation type, *Renaming*, and a single entity type, *Gene*. All entities are given, so only edge detection is required. Unlike the other tasks, the main evaluation criterion ignores the direction of the relations, so they are processed as *undirected edges *in the graph representation.

Edge detection performance was improved with external features based on two sources defining known *B. Subtilis *synonym pairs: The UniProt *B. Subtilis *gene list "bacsu" [39] and *Subti*Wiki [40], the *B. Subtilis *research community annotation wiki.

For the 300 renaming relations in the REN training data, the synonym pair was found from the UniProt list in 66% (199 cases), from *Subti*Wiki in 79% (237 cases) and from either resource in 81.3% (244 cases). For the corresponding negative edge examples, UniProt or *Subti*Wiki synonym pairs appeared in only 2.1% (351 out of 16640 examples).

At 87.0% F-score our system had the highest performance out of the three participants, exceeding the next highest system by 17.1 percentage points. If UniProt and *Subti*Wiki features are not used, performance on the development set is still 67.85%, close to the second highest performing system on the task.

### Learning curves

Moving forward after the Shared Task, it is important for the community to know how best to focus our resources on improving event extraction performance. Event extraction systems may benefit from additional optimization and extraction strategies, but on the other hand, many competing approaches have led to roughly similar performance in the BioNLP'09 and BioNLP'11 Shared Tasks.

**Figure 4:**
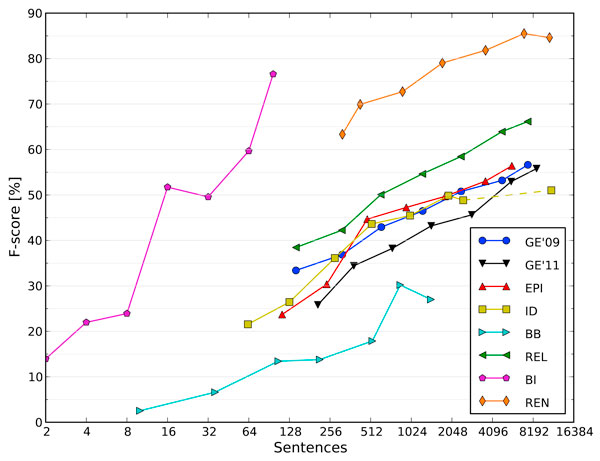
**Learning Curves**. The learning curves provide an analysis of system performance relative to dataset size. The dotted line shows the addition of GE training data to ID training data. The x-axis is binary logarithmic, and the training corpus size roughly doubles between most points in the curves (2, 4, 8, 16, 32, 64 and 100%). Thus, a linear growth in F-score indicates a need for a corresponding exponential increase in dataset size.

One source of improvement could simply be additional annotated text. However, annotation is a costly and difficult process. To determine the likely benefit from further training data, we construct learning curves for all BioNLP'11 Shared Task corpora, using our event extraction system (see Figure 4).

Learning curves are made by consecutively reducing the training set size. Our system operates on individual sentences, but in the corpora these sentences are usually grouped into documents, often consisting of a related set such as an abstract. Sentences within a single document may overlap in content, so to ensure a realistic reduction in training data, entire documents are removed at all steps [41].

Machine learning systems often show a logarithmic response to training dataset size. In the Shared Task corpora, the number of documents can however be quite small, usually in the range of a few hundreds. Thus, taking e.g. 1/1000th of the data would not be feasible. Therefore, to produce curves that clearly show the impact of the dataset size, we use a binary logarithmic scale, roughly doubling dataset size at each step. All results are predicted for the full development set, using the official Shared Task evaluation metrics.

We can see from the learning curves that generally a doubling of dataset size is required to maintain a consistent increase in F-score, indicating diminishing gains from more annotated data. However, most corpora show increased performance even at the final points of the learning curve, so some performance could still be gained by additional annotation, if enough data is added.

The BioNLP'11 corpora are of very different sizes (see Table 1). Especially the two bacteria corpora, BB and BI, are very small. The learning curves of these two show considerable variance and in some cases a reduction of training data can even result in an increase in performance. As we know that on the next smallest ID task performance is limited by training dataset size (see section *Infectious Diseases (ID)*), further development of the BB and BI extraction targets likely depends on more annotated data becoming available.

For the GE 2009 and 2011 learning curves, we have used the primary task 1 measure. Of all tasks, only these two have a directly comparable evaluation metric. It seems that overall, the old GE 2009 corpus is slightly easier to learn, a result consistent with the inclusion of more heterogeneous full-text articles in the 2011 corpus. However, when dataset size increases, performance seems to converge, and when using 64-100% of the data, performance is very similar for both corpora.

### Self-training

Self-training is a machine learning technique in which a suitable subset of a system's output is used as additional training data for the same system. In the domain of biomedical NLP, self-training was successfully applied for instance to syntactic parsing [16] and word sense disambiguation [42]. We tested the effect of self-training on the GE task (subtask 1), using data from EVEX, a publicly available database of automatically extracted events produced by applying our BioNLP'09 Shared Task system to the entire 2009 distribution of PubMed citation titles and abstracts [10,43].

Typically, self-training examples are selected based on their confidence score assigned by the system during extraction. Low-confidence examples are avoided since they have a higher proportion of false positives and would thus not be likely to provide useful training data. Very high confidence events, on the other hand, may not provide sufficiently new information, as the system is already able to extract them reliably. To test the effect of event confidence on its usability as self-training data, we first renormalize the confidence scores of all events in EVEX to *μ *= 0 and *σ *= 1, i.e. zero mean with standard deviation one. Having observed that the mean event confidence score in EVEX differs substantially depending on the type of the event, the number of entity arguments, and the number of recursive event arguments, we normalize each subset of EVEX events defined by these three criteria separately. We then select four sets of EVEX events for self-training, based on how many standard deviations above or below the mean their normalized confidence score is. We randomly select 20,000 EVEX events for each of the four sets: set *S*_0 _contains events with confidence in the range [-0.5, 0.5), set S_1 _events with confidence in the range [0.5, 1.5), and so forth for sets *S*_2 _and *S*_3_.

For each of the four self-training sets, we measure the performance of the system, with the set included in the training data, and compare it to the baseline performance where no self-training data is used. The results are presented in Table 4. The self-training performance surpasses that of the baseline for sets *S*_2 _and *S*_3_, however the overall gain of 0.7 pp (for *S*_2_) is only very modest and does not manifest on the test set, where the overall F-score decreases by 0.12 pp when self-training is used.

**Table 4:** Results of self-training

	Random distribution (devel/test)	Even distribution (devel/test)
*S*_3_	55.97%	56.17%
*S*_2_	56.18%/52.72%	56.83%/53.21%
*S*_1_	54.83%	55.78%
*S*_0_	55.67%	55.79%

baseline	55.46%/52.84%	55.46%/52.84%

Performance of the system on the GE subtask 1 in terms of F-score on the overall *Approximate Span & Recursive matching *criterion. Random distribution refers to self-training example selection by random sampling, whereas even distribution refers to selection of equal amount of examples for each event type and argument combination. Baseline is the performance of the system with no self-training (trained on GE subtask 1 data only).

In a follow-up experiment, we focus on the fact that the distribution of events is very uneven. First, most events only have a single *theme *argument and second, event types such as *protein catabolism *are considerably more rare than for example *regulation*. This naturally also reflects in the randomly selected self-training sets, which provide little additional data for rare event types and argument combinations. We thus tested a sampling strategy where for each of the 22 combinations of event type, number of entity arguments, and number of event arguments, we sampled a maximum of 2,000 event examples in the confidence range [1.5, 2.5), i.e. the range that gave best results in the previous experiment. In addition, for each of these events, we also include all their recursively nested events so as to preserve event structures in their entirety. In total, the self-training set comprised 54,270 events. This strategy resulted in an increase in F-score of 1.4 pp (from 55.46% to 56.83%) on the development set and 0.4 pp (from 52.84% to 53.21%) on the test set, for GE subtask 1, and is thus a clearly better strategy than a simple random sampling. Detailed results are shown in Table 5, however, there is no obvious pattern as to which event classes benefit from self-training, likely to some extent due to the small magnitude of the overall gain.

**Table 5:** Detailed results of the even distribution self-training experiment

Event type	#	**freq**.	Baseline [%]	ST [%]	Δ (devel.)	Δ (test)
Gene expression	749	23.1%	78.79	79.21	+0.42	+0.50
Transcription	158	4.9%	59.78	61.71	+1.93	-0.33
Protein catabolism	23	0.7%	89.80	95.83	+6.03	-6.32
Phosphorylation	111	3.4%	85.97	86.49	+0.52	+0.46
Localization	67	2.1%	64.91	66.67	+1.76	+6.00
Binding	373	11.5%	51.30	50.88	-0.42	-0.61
Regulation	292	9.0%	38.28	38.33	+0.05	+1.16
Positive regulation	999	30.8%	42.74	47.14	+4.40	+1.70
Negative regulation	471	14.5%	41.37	42.16	+0.79	-3.04

Overall	3,243	100.0%	55.46	56.83	+1.37	+0.37

Performance of the system on the GE subtask 1 in terms of F-score on the overall *Approximate Span & Recursive matching *criterion. Baseline and self-training (ST) results, as well as evaluation event counts are given for the development set. Difference (Δ) in F-score is given for both the development and test sets.

These results are obtained when both training and evaluating the system on GE subtask 1 only. Combined training for subtasks 1 and 2 gives a subtask 1 performance of 53.30% on the test set, the official result of the system in the Shared Task. This performance is 0.1 pp higher than the 53.21% obtained with self-training on GE subtask 1 only. Further preliminary experiments with self-training for combined GE subtasks 1 and 2 had so far only a negligible effect on the performance.

While the magnitude of the performance differences does not allow too firm conclusions to be drawn, it is clear that with appropriate selection strategy, self-training does have the potential for a performance gain, as shown both on the development and test sets. With a PubMed-wide event resource with nearly 20 million events easily available, it is a direction certainly worth further investigation regarding which exact subset of events to include as self-training data to maximize the gain.

## Conclusions

We have developed a system that addresses all tasks and subtasks in the BioNLP'11 Shared Task, with top performance in several tasks. With the modular design of the system, all tasks could be implemented with relatively small Modifications to the processing pipeline. The graph representation which covered naturally all different task annotations was a key feature in enabling fast system development and testing. As with the Turku Event Extraction System developed for the BioNLP'09 Shared Task, we release this improved system for the BioNLP community under an open source license at bionlp.utu.fi.

Of all the tasks, the GE-task, which extends the BioNLP'09 corpus, is best suited for evaluating advances in event extraction in the past two years. For the GE'09 corpus, in the BioNLP'09 Shared Task we achieved a performance of 51.95% (shortly afterwards improved to 52.86%) and in 2010 Miwa et. al. reached 56.00% [7,8]. Comparing our current system's performance on the GE'09 corpus with the GE'11 one, we can assume that the two corpora are of roughly equal difficulty. Therefore we can reason that since the BioNLP'09 Shared Task, event extraction performance has increased about four percentage points, the highest performance on the 2011 GE-task being 56.04% by team FAUST. It appears that event extraction is a hard problem, and that the immediate performance gains have already been found. We hope the BioNLP'11 Shared Task has focused more interest in the field, hopefully eventually leading to breakthroughs in event extraction and bringing performance closer to established BioNLP fields such as syntactic parsing or named entity recognition.

That our system could be generalized to work on all tasks and subtasks, indicates that the event extraction approach can offer working solutions for several biomedical domains. A potential limiting factor currently is that most task-specific corpora annotate a non-overlapping set of sentences, necessitating the development of task-specific machine learning models. Training on multiple datasets could mean that positives of one task would be unannotated on text from the other task, confusing the classifier. On the other hand, multiple overlapping task annotations on the same text would permit the system to learn from the interactions and delineations of different annotations. System generalization has been successfully shown in the BioNLP'11 Shared Task, but has resulted in a number of separate extraction systems. It could well be that the future of event extraction requires also the generalization of corpus annotations.

Our results on self-training demonstrate that system output can be used to improve performance in some cases. Self-training is a promising direction for system improvement, as in addition to performance improvements, it might produce a system more suited for use with heterogeneous real-world data. Our continued efforts on PubMed-scale event extraction will in the future provide more data for researchers interested in self-training for event extraction.

As future directions, we will continue to improve the scope and performance of the Turku Event Extraction System. We are continuing our work on PubMed-scale event extraction and the EVEX dataset, and will use for this project several of the new extraction targets introduced by the BioNLP'11 Shared Task.

## Competing interests

The authors declare that they have no competing interests.

## Authors' contributions

JB designed and implemented the upgrade of the Turku Event Extraction System for the BioNLP 2011 Shared Task. FG and JB designed and implemented the learning curve and self-training analyses. JB and FG have drafted the manuscript. TS supervised the study. All authors have read and approved the final manuscript.
